# Estimating and Interpreting Effects from Nonlinear Exposure-Response Curves in Occupational Cohorts Using Truncated Power Basis Expansions and Penalized Splines

**DOI:** 10.1155/2017/7518035

**Published:** 2017-09-20

**Authors:** Elizabeth J. Malloy, Jay M. Kapellusch, Arun Garg

**Affiliations:** ^1^Department of Mathematics and Statistics, American University, Washington, DC, USA; ^2^Occupational Science & Technology, University of Wisconsin-Milwaukee, Milwaukee, WI, USA

## Abstract

Truncated power basis expansions and penalized spline methods are demonstrated for estimating nonlinear exposure-response relationships in the Cox proportional hazards model. R code is provided for fitting models to get point and interval estimates. The method is illustrated using a simulated data set under a known exposure-response relationship and in a data application examining risk of carpal tunnel syndrome in an occupational cohort.

## 1. Introduction

The Cox proportional hazards (PH) model is frequently used to model survival data or time-to-event data, particularly in the presence of censored survival times [1]. The hazard, or instantaneous risk, of an event of interest, typically mortality or morbidity, is modeled in terms of one or more explanatory variables relative to an unspecified baseline hazard rate. This hazard ratio (HR) for the outcome—often interpreted as a type of relative risk—is the effect of interest and may be used in epidemiological studies for risk assessment. In occupational settings, it is common to have an occupational exposure as one of the explanatory variables in the model and the association between the outcome and this exposure is of interest. In this case, the HR, or its logarithm, may be referred to as the exposure-response relationship. The focus is thus on estimation of and inferences for this exposure-response relationship. Nonlinear exposure-response relationships do arise in the analysis of occupational cohorts [2–7]. An attenuation of the HR at the highest exposures has been well documented [8] and interpretation of nonlinear exposure-response relationships is useful in epidemiological risk assessment [9]. Methods for modeling nonlinearities are needed in those situations when a linear exposure-response is not expected or when one desires to formally assess a nonlinear association.

Consider an occupational cohort with *i* = 1,…, *n* individuals on which the time until a given health event of interest, *t*_*i*_, is measured. These times may be right censored if the individual did not have the event of interest during the study time. This is denoted by an indicator variable,* c*_*i*_, which takes the value of 1 if the individual had the event and 0 if the time is censored. The general form of the Cox PH model for a single covariate is

(1) $$\begin{array}{c} \lambda \left(\;t\;|\;x_{i}\;\right)=\lambda_{0}\left(\;t\;\right)exp\left[\;\beta x_{i}\;\right], \end{array}$$

where *λ*(*t*∣*x*_*i*_) is the hazard function, *x*_*i*_ is the corresponding quantitative exposure variable, *λ*_0_(*t*) is the baseline hazard function, and *β* is the regression coefficient. In this form, the logarithm of the hazard ratio (HR) is linear, *βx*_*i*_, and the exposure-response relationship is described as linear (on the log-scale). The HR for a given exposure* x *is exp[*βx*], where exp[*β*] is interpreted as a multiplicative effect when comparing the hazard (or risk) at exposures one unit apart.

A nonlinear exposure-response relationship can be modeled by including a transformation of* x*_*i*_ in the model:

(2) $$\begin{array}{c} \lambda \left(\;t\;|\;x_{i}\;\right)=\lambda_{0}\left(\;t\;\right)exp\left[\;s\left(\;x_{i}\;\right)\;\right], \end{array}$$

where *s*(·) is a known function. Many user-specified choices exist for this functional form, such as exposure categories and algebraic functions [10]. These methods generally require user input for exposure category cut-points or the algebraic expression, such as a logarithmic transformation of the exposure variable, *x*. An alternative to this type of specification is to use methods which do not impose a priori shape or categorical constraint on the exposure-response relationship. Examples of such “smoothing” methods are polynomial regression splines [11] and penalized splines [12]. One criticism of smoothing methods is their lack of interpretable parameters [13], such as the *β* regression coefficient. Nevertheless, interpretable estimates (i.e., HR) with corresponding confidence intervals can be found directly from the fitted model, even when using smoothing methods. We illustrate this interpretation and the use of these methods (regression and penalized splines) and compare them to exposure categories and standard algebraic forms in the context of occupational physical exposure analyses.

This manuscript provides a detailed introduction to modeling and interpreting nonlinear exposure-response curves using these spline functions. We assume familiarity with the Cox PH model and survival data. The remainder of the paper is structured in three sections.  Section 2 gives the theoretical Cox proportional hazards model for spline-based estimates of nonlinear exposure-response associations. These methods are simultaneously explained and illustrated using a simulated data set under a known nonlinear exposure-response relationship. The section ends with an examination of the interpretation of the estimated HR using point estimates and pointwise confidence intervals.  Section 3 gives an application in which we examine the nature of the association between job physical demands and incidence of carpal tunnel syndrome (CTS) in an occupational cohort of 569 individuals previously analysed by Garg et al. [14]. Final discussion and comments are in Section 4. An Appendix contains additional theoretical details for estimation and inferences. The R software [15] code is available from the corresponding author.

## 2. The Cox Proportional Hazards Model for a Nonlinear Exposure-Response Relationship

### 2.1. Splines and the Cox Proportional Hazards Model

In the Cox PH model in (2), we use a basis expansion representation of the exposure-response function *s*(*x*_*i*_) based on a linear combination of known basis functions, *f*_*j*_(*x*_*i*_),

(3) $$\begin{array}{c} s\left(\;x_{i}\;\right)=\overset{J}{\underset{j=1}{\sum }}b_{j}f_{j}\left(\;x_{i}\;\right). \end{array}$$

There is vast literature on using basis functions in linear models and there are many options for selecting basis functions to use. The text by Ruppert et al. [16] provides many nice examples. A simple basis for a linear exposure-response relationship would consist of the single function*f*_1_(*x*_*i*_) = *x*_*i*_. For a quadratic association, the basis functions are *f*_1_(*x*_*i*_) = *x*_*i*_ and *f*_2_(*x*_*i*_) = *x*_*i*_^2^. This can be extended to a polynomial of degree* p* by using the* p* basis functions {*x*_*i*_, *x*_*i*_^2^, *x*_*i*_^3^,…, *x*_*i*_^*p*^}. Note that we omit the unit basis function, which corresponds to the intercept term in the model, because in the Cox PH model setting the intercept is subsumed by the unspecified baseline hazard function. Estimates in the Cox PH model are relative to the unspecified baseline hazard.

To provide flexibility in capturing local features in the exposure-response curve, polynomial spline terms may also be used as basis functions. A spline function is a function, typically a polynomial, defined on a subinterval of the range of exposures. Splines allow for estimation of the exposure-response relationship using a piecewise-defined curve. They are generally considered to provide more flexibility in estimating nonlinear relationships than polynomials or other algebraic functions. To define a piecewise linear curve over four regions in which the slope changes from region to region, we would use a set of basis functions consisting of the functions {*x*_*i*_, (*x*_*i*_ − *k*_1_)_+_, (*x*_*i*_ − *k*_2_)_+_, (*x*_*i*_ − *k*_3_)_+_}, where {*k*_1_, *k*_2_, *k*_3_} are exposure values at which the slope changes and are called “knots.” These are user-specified values, similar in spirit to categorical cut-points where changes in the response occur. The “+” subscript notation indicates the function is equal to the expression given in parentheses when that expression is positive. That is, (*x* − *k*_1_)_+_ = *x* − *k*_1_if *x* > *k*_1_ and 0 otherwise. In this way, a nonlinear association can be estimated by fitting the model in (2) with *s*(*x*_*i*_) = *b*_1_*x*_*i*_ + *b*_2_(*x*_*i*_ − *k*_1_)_+_ + *b*_3_(*x*_*i*_ − *k*_2_)_+_ + *b*_4_(*x*_*i*_ − *k*_3_)_+_. The standard maximum partial likelihood method yields estimates of the coefficients, giving an estimated ln(HR) of $\hat{s}\left(x_{i}\right)={\hat{b}}_{1}x_{i}+{\hat{b}}_{2}{\left(x_{i}-k_{1}\right)}_{+}+{\hat{b}}_{3}{\left(x_{i}-k_{2}\right)}_{+}+{\hat{b}}_{4}{\left(x_{i}-k_{3}\right)}_{+}$. Higher order (degree) polynomials can also be used by expanding the set of basis functions to include all polynomial terms up to degree* p* and then* K *degree* p* spline functions, defined using* K* knots: {*x*_*i*_, *x*_*i*_^2^, *x*_*i*_^3^,…, *x*_*i*_^*p*^, (*x*_*i*_ − *k*_1_)_+_^*p*^, (*x*_*i*_ − *k*_2_)_+_^*p*^,…, (*x*_*i*_ − *k*_*K*_)_+_^*p*^}. This set is called the truncated power basis of degree* p* [16] and allows for smoother exposure-response estimates as functions formed from linear combinations of these basis functions have *p* − 1 continuous derivatives. With small to moderate numbers of knots, a standard Cox PH model can be fit to estimate the nonlinear exposure-response curve.

**Figure 1:**
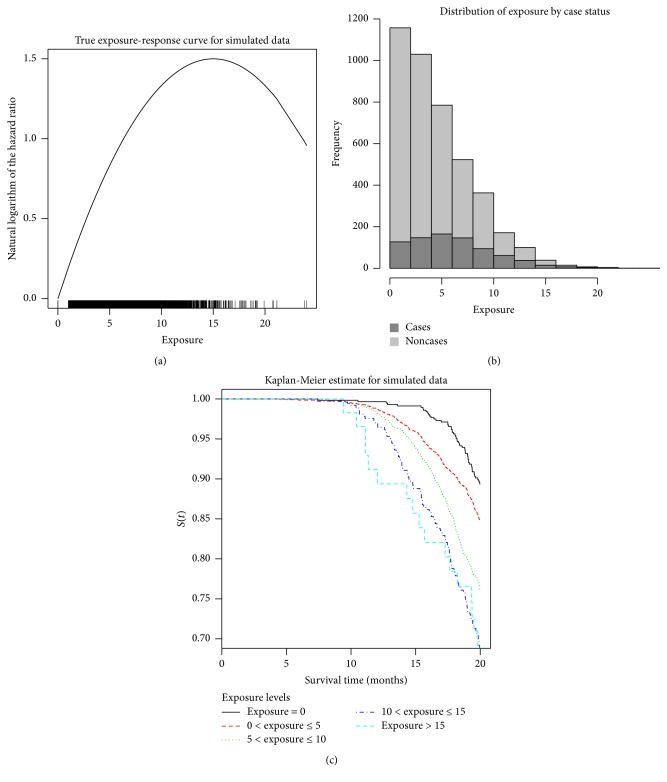
True exposure-response relationship used to simulate data (a). Histogram of the simulated exposure data (b). Kaplan-Meier estimates of the survival functions for five exposure groups (c).

As an illustration, we simulated a data set of *n* = 5000 individuals whose exposure-response relationship shows an attenuation at the highest exposures; see Figure 1. Specifically, on the log-scale, the true *s*(*x*) is a quadratic function with a maximum at an exposure of *x* = 15 units. These data were generated using the method described in Bender et al. [17] and Malloy et al. [18]. The exposure variable was set so that approximately 13% of individuals were unexposed. With this exposure distribution (displayed in Figure 1) and the corresponding true exposure-response relationship, approximately 16% of individuals are cases. Survival times were left skewed with the median case survival time approximately 17 time-units and the median for noncases about 20 time-units. To give a sense of how survival varies with exposure in this simulated data set, prior to fitting the Cox PH models, we created five equally spaced exposure categories and found the estimated survival functions using the Kaplan-Meier estimate using the survival package [19] in R. The five exposure categories were a baseline group with no exposure (approximately 13% of observations), those with exposures between 0 and 5 (approximately 46% of observations), between 5 and 10 (32%), between 10 and 15 (8%), and above 15 (1%).  Figure 1(c) shows the estimated survival functions for these five exposure categories. The baseline/no exposure group has the highest survival rates while the highest exposed group has the lowest survival rates, up until a survival time of about 15 time-units, at which point the highest exposed group overlaps with the 10- to 15-exposure group. This is consistent with the generating model, in which there is a drop in the logarithm of the hazard ratio for these highest exposed individuals (Figure 1(a)).

**Figure 2:**
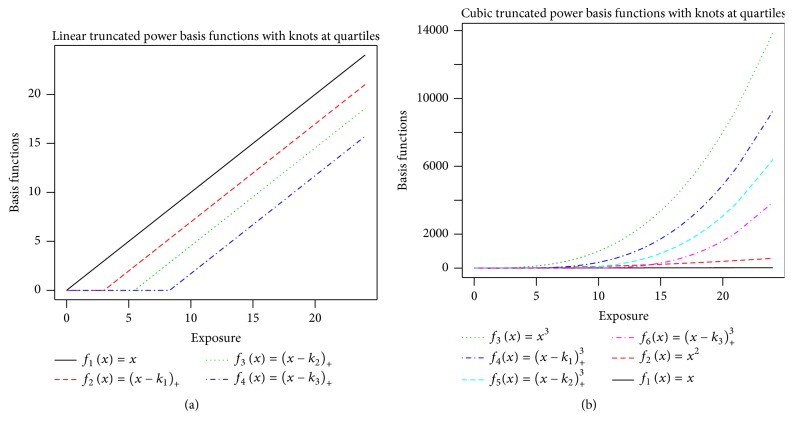
Linear spline (a) and cubic spline (b) basis functions using knots at quartiles of the case exposures (*k*_1_ = 3.0,* k*_2_ = 5.5, and* k*_3_ = 8.3).

We illustrate the spline-based methods for estimating the exposure-response relationship,* s*(*x*), which is the logarithm of the hazard ratio (ln(HR)). Using a linear truncated power basis with three knots requires four basis functions, *f*_1_(*x*) = *x*, *f*_2_(*x*) = (*x* − *k*_1_)_+_, *f*_3_(*x*) = (*x* − *k*_2_)_+_, and *f*_4_(*x*) = (*x* − *k*_3_)_+_.  Figure 2(a) displays these four functions when the knots were chosen to be at the quartiles of the exposure distribution of the cases (*k*_1_ = 3.0,* k*_2_ = 5.5, and* k*_3_ = 8.3). A cubic truncated power basis representation using these same knots requires six basis functions, *f*_1_(*x*) = *x*, *f*_2_(*x*) = *x*^2^, *f*_3_(*x*) = *x*^3^, *f*_4_(*x*) = (*x* − *k*_1_)_+_^3^, *f*_5_(*x*) = (*x* − *k*_2_)_+_^3^, and *f*_6_(*x*) = (*x* − *k*_3_)_+_^3^ (Figure 2(b)).

Fitting the Cox PH model requires using the basis function transformations of the exposure variables as the covariates in the model (and introduces regression coefficients* b*_*j*_),

(4) λt ∣ xi=λ0texp⁡b1xi+b2xi−k1++b3xi−k2++b4xi−k3+

for the linear truncated power basis model and

(5) λt ∣ xi=λ0texp⁡b1xi+b2xi2+b3xi3+b4xi−k1+3+b5xi−k2+3+b6xi−k3+3

for the cubic truncated power basis model. Standard model fitting methods are used for the Cox PH model (i.e., maximum partial likelihood) to obtain the estimates of the coefficients and hence of the exposure-response curve,

(6) s^x=b^1x+b^2x−k1++b^3x−k2++b^4x−k3+,s^x=b^1x+b^2x2+b^3x3+b^4x−k1+3+b^5x−k2+3+b^6x−k3+3

for the linear and cubic truncated power basis models, respectively. For our simulated cohort example, these estimates after rounding the coefficients to two decimal places are

(7) s^x=0.13x+0.03x−3.0+−0.07x−5.5+−0.06x−8.3+,s^x=0.19x−0.04x2+0.01x3−0.02x−3.0+3+0.01x−5.5+3−0.01x−8.3+3.

The estimated HR can be found simply by exponentiating, $\hat{HR}=\exp\left[\hat{s}\left(x\right)\right]$. Note that this is the estimated hazard at a given exposure,* x*, relative to the baseline hazard, generally corresponding to* x* = 0 (i.e., unexposed).

The R software package used here for fitting Cox PH models and obtaining the estimates is the survival package [19]. The predict() function in this package uses the mean exposure value as the reference category for these estimated hazard ratios. When there is a single covariate entered as a linear term, using $\overset{-}{x}$ as the reference value is reasonable as it provides a comparison of the estimated hazard at a given exposure relative to the “typical” (i.e., mean) exposure in the cohort. Often other exposure values may be the desired reference. In particular, using no exposure as the reference is also meaningful in the context of occupational hazards when we want to compare the estimated hazard of death or a health outcome at a given occupational exposure level to the hazard when not exposed. Furthermore, when multiple covariates are entered, such as the four covariates needed for the linear truncated power basis, this mean reference value is taken with respect to each covariate entered into the model. That is, with the four covariates defined as *x*_1_ = *x*, *x*_2_ = (*x* − 3.0)_+_, *x*_3_ = (*x* − 5.5)_+_, and *x*_4_ = (*x* − 8.3)_+_, then a side effect of the predict() function in R is the hazard ratio and is computed with respect to ${\overset{-}{x}}_{1}$, ${\overset{-}{x}}_{2},{\overset{-}{x}}_{3},\text{and}\;{\overset{-}{x}}_{4}$, which in this context are the mean values of the basis functions averaged over all individuals in the data set. This has no meaningful interpretation for basis function estimates.  Appendix A gives the mathematical details for computing the estimated HR and ln(HR) with any user-chosen exposure as the reference based on the output from the Cox PH model fit in the survival package. It does so for general linear combinations of coefficients in a Cox PH model but is specifically applied to the basis expansion context given here. The corresponding R scripts for the linear truncated power basis expansion are displayed in Appendix B.

**Figure 3:**
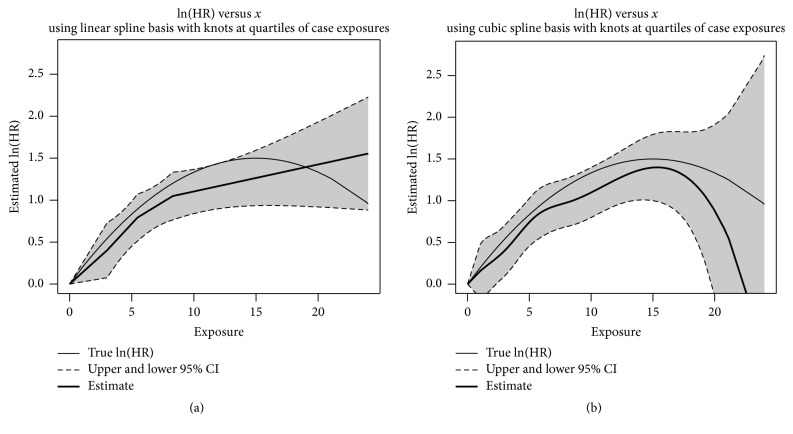
Estimated ln(HR) and corresponding pointwise 95% confidence intervals using linear spline (a) and cubic spline (b) basis functions with knots at quartiles of the case exposures (*k*_1_ = 3.0,* k*_2_ = 5.5, and* k*_3_ = 8.3).

Based on the calculations and code in Appendices A and B, respectively, the plots in Figure 3 illustrate the estimated exposure-response relationship using *x* = 0 as the reference point for the ln(HR). Both the linear and cubic truncated power basis expansions are illustrated along with pointwise 95% confidence intervals at each exposure value in the data set. For this simulated data set, both truncated power bases follow the general trend of increasing relative hazard up until an exposure of 15 units. In this example, the estimate using a linear truncated power basis always increases, contrary to the true exposure-response curve. Conversely, the estimate using the cubic truncated power basis starts to decrease after about *x* = 15.3 units, although it underestimates the ln(HR) relative to the true exposure-response curve. Both truncated power bases' 95% pointwise confidence interval curves essentially contain the true curve, except for a region between about* x *= 11.4 and *x* = 12.4 for the linear truncated power basis.

### 2.2. B-Spline Basis Functions and Penalized Fits

**Figure 4:**
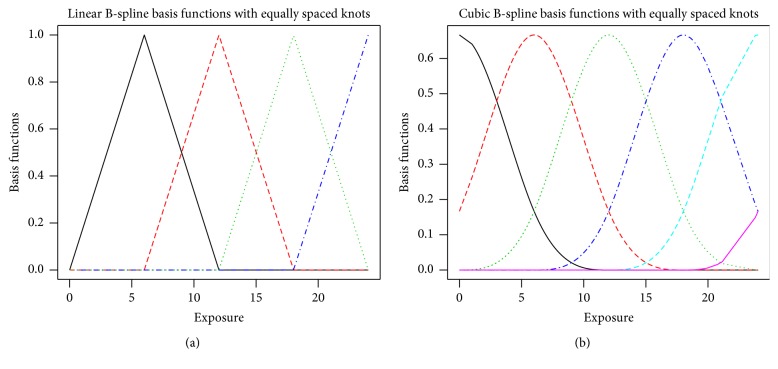
Linear B-spline (a) and cubic B-spline (b) basis functions using equally spaced knots.

Although the truncated power basis functions are relatively easy to visualize and implement, they do require a choice of the polynomial degree* p*, the number of basis functions* K* +* p*, and the locations of the knots. Smoother (continuously differentiable) estimates are found with higher degree; however, these models may become numerically unstable. Alternative piecewise-defined polynomials, called B-splines, overcome this numerical instability. B-splines are defined recursively through lower degree spline functions using an algorithm given in de Boor [20] with further details of their properties given in Eilers and Marx [21].  Figure 4 illustrates linear (a) and cubic (b) B-spline basis functions. Both were created using equally spaced knots but any knots can be specified to define the basis functions. The scale of the vertical axis is substantially reduced as compared to the axes for the truncated power basis functions in Figure 2, thus substantially improving numerical stability.

**Figure 5:**
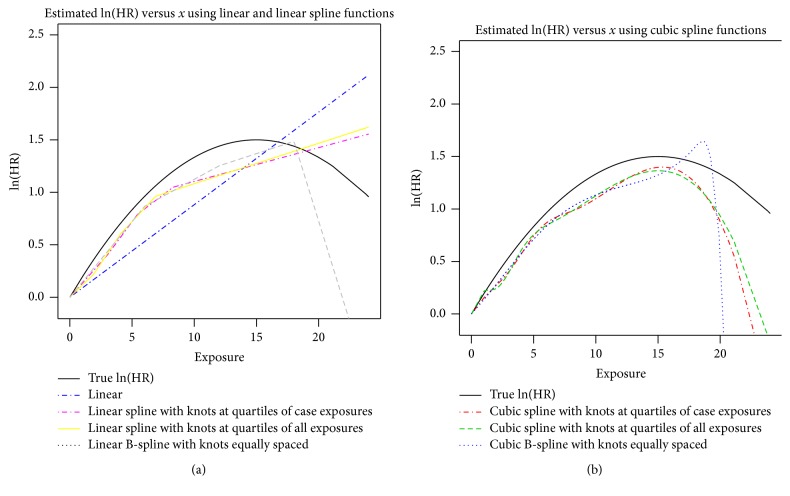
Estimated exposure-response curves on the natural logarithmic scale (logarithm of the hazard ratio) using truncated power basis functions and B-spline basis functions.

With the knots and degree specified, the B-spline basis functions are then the known functions *f*_*j*_(*x*) used in the basis expansion representation of the exposure-response curve *s*(*x*) in (3) above and model fitting may proceed as described in the previous section. Cubic B-splines are a reasonable choice for smooth estimates; however, these estimates may be sensitive to user-selected knot choice. For example, in Figure 5, the estimate using linear B-spline bases with equally spaced knots shows a decrease in the ln(HR) after an exposure of about *x* = 18.0, whereas the linear B-spline with knots at quartiles does not. A large number of evenly spaced basis functions can reduce dependency of user-specified knots but may also result in overfitting or “noisy” estimates. Penalized splines (psplines, [21]) address this problem by combining the B-spline basis expansion and a penalized fit that balances the need for flexibility of exposure-response shape against fitting of noise in the data.

Penalized estimates for the unknown parameters in the basis expansion (3) are found by maximizing a penalized log partial likelihood, *l*(*b*) − *θP*(*b*), where *l*(*b*) denotes the log partial likelihood function for the Cox PH model [1], *b* is the vector of coefficients (*b*_1_,…, *b*_*J*_) in (3), *P*(*b*) is an expression which restricts or penalizes the size of these coefficients, and *θ* is a user-specified or data-estimated tuning parameter which controls the degree of smoothing. A typical penalty term places a constraint on the curvature of the estimate of *s*(*x*) via its second derivative:

(8) $$\begin{array}{c} l\left(\;b\;\right)-\theta \int {\left[\;s^{\prime \prime }\left(\;x\;\right)\;\right]}^{2}dx. \end{array}$$

The smoothing parameter *θ* in (8) is related to the degrees of freedom (df), or effective number of parameters, associated with the estimate $\hat{s}\left(x\right)$. Having no penalty (*θ* = 0) results in all *J* terms in the basis expansion in (3) being used with their corresponding* J* coefficients being completely unconstrained, thus giving df = *J*. Given the penalty on the curvature of the estimate of *s*(*x*), as *θ* approaches infinity the df approaches 1, corresponding to a linear term for the exposure variable, *s*(*x*) = *βx* [12]. Thus for values of *θ* between 0 and infinity, the degrees of freedom are 1 ≤ df ≤ *J*. Data-driven methods are frequently used to select the degrees of freedom (or smoothing parameter). Methods such as the Akaike information criterion (AIC) [22] and an adjusted version of this called the corrected AIC (AICc) [23] are included in the pspline() function in the R survival package [19].

As with the truncated power basis expansion method of Section 2.1, the default predicted HR or ln(HR) in R is mean-centered relative to each covariate in the model; thus without adjustment these estimates are completely meaningless when using basis expansion methods. The methods in the Appendices can be used with penalized spline fits to obtain meaningful estimated HR values or ln(HR) values with a user-specified reference exposure. We opt to use a cubic B-spline basis as these provide reasonably smooth estimates and are the default choice in the pspline() function. We also use automatic selection of the degrees of freedom using the AICc method and the default setting for the number of spline terms (nterm = 15) in the pspline() function in R. Note that this default corresponds to 17 actual basis functions in the expansion (after dropping one as it is equivalent to the redundant constant term subsumed by the baseline). We use this same setting (nterm = 15) even when preselecting the desired degrees of freedom (the default is nterm = 2.5∗df).

**Figure 6:**
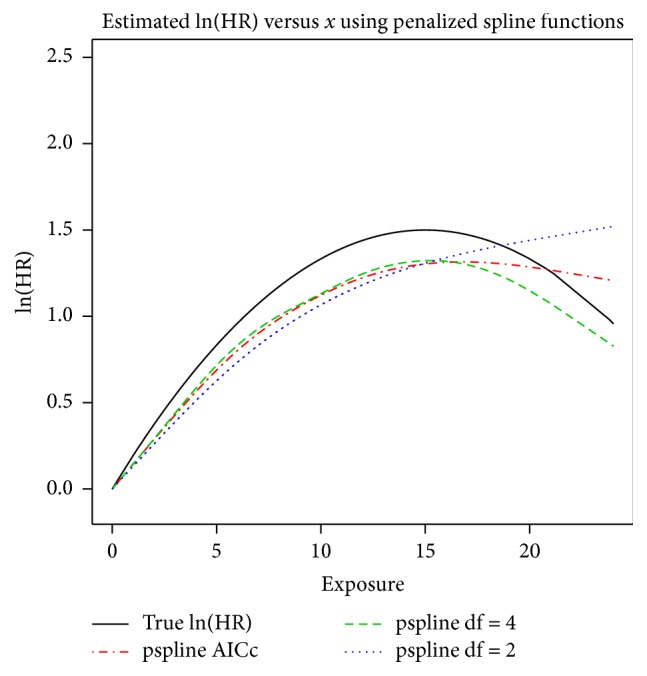
Estimated exposure-response curves on the natural logarithmic scale (logarithm of the hazard ratio) using penalized splines.

To illustrate penalized estimates, we used our simulated data with the known quadratic nonlinear exposure-response curve. We fit penalized splines as described above, under three conditions: with df selected using AICc, with df = 2, and with df = 4. The estimates using an unexposed reference are displayed in Figure 6 along with the corresponding true exposure-response relationship. The AICc method chose df = 2.9 and all three estimates indicate an increasing risk up until approximately *x* = 15 for df = 4, *x* = 16.75 for df = 2.9, and continuing to increase for df = 2.

### 2.3. Interpretation of Estimates

Table 1 gives estimated hazard ratios at exposure values approximately equal to 2.0, 3.0, 4.0, 5.0, 7.0, 9.0, 19.3, 21.1, and 24.0. These roughly correspond to the quartiles of noncase exposures (1.8, 3.8, and 6.6), the quartiles of case exposures (3.0, 5.5, and 8.3), and the maximum overall case exposure (19.3). The two additional values correspond to higher exposures in the region where the true exposure-response relationship attenuates and data become sparse.

These estimated hazard ratios give the estimated hazard (risk) of the outcome at a given exposure relative to the hazard when unexposed. For instance, we estimate from the penalized spline fit using AICc that the hazard of the event when exposed at a level of 2.0 is 1.3 times that when unexposed, corresponding to a 30% increase in hazard at this exposure level. For this simulated data set, the linear truncated power basis with knots at the quartiles of the case exposures and the penalized spline fit are comparable; however while the former does attenuate, it does not decrease at the highest exposure values.

### 2.4. Hypothesis Tests with Basis Function Expansions

The pspline() function in the survival package provides a chi-square test for a test of the nonlinearity in the penalized fit. We can conceive of this as a test of the null hypothesis Ho: *s*(*x*) = *bx* versus the alternative Ha: *s*(*x*) = ∑_*j*=1_^*J*^*b*_*j*_*f*_*j*_(*x*). The model fit R summary output for the penalized spline fit using the AICc to get the degrees of freedom is provided in Appendix C. From this, the test for the nonlinear component has degrees of freedom of 1.86 and a test statistic value of 11.3, giving a* p* value of 0.003. Thus, for these data the nonlinear fit is warranted. Details of this test can be found in Chapter 5 of Therneau and Grambsch [24]. Similar hypothesis tests can be performed using the truncated power basis methods. These tests are described in Appendix D.

## 3. Data Application

Garg et al. [14] examined the association between risk of carpal tunnel syndrome (CTS) and job physical exposure as measured by the strain index (SI) [25], a semiquantitative distal upper limb physical exposure quantification method. The SI method yields a numerical score that is believed to reflect strain on the distal upper limbs as a result of performing hand work. Their cohort included 429 workers from 10 predominantly manufacturing facilities in the Midwest, USA. There were 35 incident cases of CTS over the 6-year study period. Demographic and other covariates were also measured, further details of which can be found in Garg et al. [14]. We include in our analyses the same covariates in Garg et al. [14], which are age transformed using a linear spline with knot at 44.3 years, body mass index, the number of distal upper extremity musculoskeletal disorders other than CTS, rheumatoid arthritis, hobbies such as gardening, and psychosocial measures such as feelings of depression.

**Figure 7:**
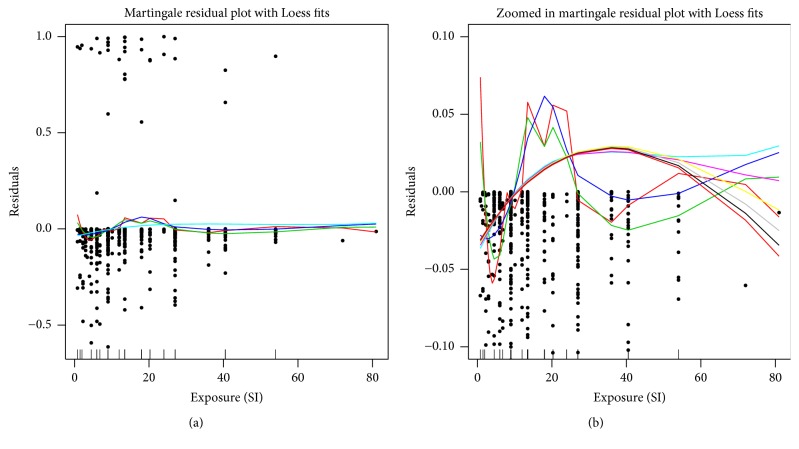
Unscaled (a) and scaled (b) plots of the martingale residuals versus exposure (SI) with Loess curves using various degrees of smoothing (0.4 to 2.0) from a Cox proportional hazards model with all covariates excluding the exposure variable. (b) is scaled to focus on the Loess curves. The distribution of the exposure variable is given in the rug plot on the *x*-axis.

An initial assessment of a nonlinear exposure-response was made using plots of the martingale residuals. To do so, the Cox PH model with all covariates excluding the exposure (SI) variable was fit and the martingale residuals were obtained. These martingale residuals were then plotted against the exposure variable and Loess curves were added to the plot. The residual plot is displayed in Figure 7 at full scale and zoomed in on the curves using smoothing parameters equally spaced from 0.4 to 2.0. Assessment of the Loess curves suggested a nonlinear exposure-response relationship for the hazard ratio of CTS with SI. Depending on the degree of smoothness chosen for the Loess, this association was quadratic or cubic in nature. The deviance residuals were also examined and showed similar results (output omitted).

**Figure 8:**
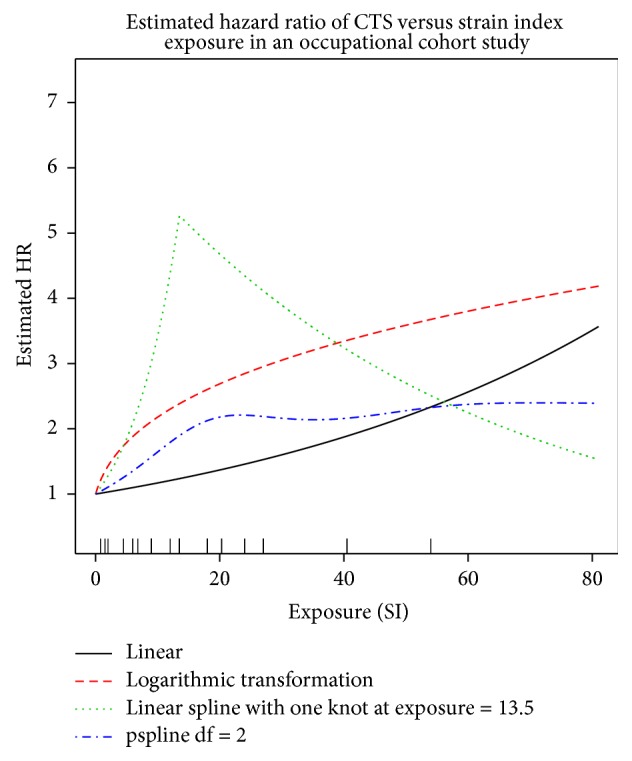
Estimated exposure-response curves for carpal tunnel syndrome and strain index in a cohort of 569 workers. Rug plot is of cases.

To address the nonlinearity displayed in the residual plots, four models were examined for these revisited analyses: two parametric functional forms (linear and a logarithmic transformation), a linear spline function with a single knot at the median exposure of SI = 13.5 units (as in [14]), and a penalized spline fit with 2 degrees of freedom. These models had similar AIC values that ranged from a minimum of 372.2 (the linear spline with knot at 13.5) to a maximum of 374.6 (the linear). Estimated fits from these models are displayed in Figure 8 and suggest an increase in the hazard ratio for exposures up to 13.5 or more, depending on the model examined. At these exposures, the spline models suggest a decline in the hazard ratio (the linear spline) or a tapering off (the pspline) of risk at the upper exposure levels, whereas the parametric linear and logarithmic transformations both suggest increasing hazard ratio with increasing risk, with the logarithmic estimating a higher risk than the linear transformation.

Table 2 gives estimated hazard ratios and corresponding confidence intervals at exposure values equal to 0.8, 6.0, 9.0, 13.5, 18.0, 20.3, and 54.0. These correspond to the quartiles of noncase exposures (6.0, 9.0, and 18.0), the quartiles of case exposures (9.0, 13.5, and 20.3), and the minimum and maximum overall case exposures (0.8 and 54.0, resp.). All estimated hazard ratios are elevated at these exposures (HR > 1.0) although many of the 95% confidence intervals do contain HR = 1.0, indicating nonsignificant effects at a 5% significance level if one considers a two-sided hypothesis test of HR = 1.0. The confidence intervals are widest for the logarithmic and linear spline models. They are also relatively wider at the highest exposures for all models, which is not surprising when we examine the distribution of case exposures, as given on the *x*-axis of Figure 8. This indicates the sparseness of cases at higher exposures and is reflected by the uncertainty in the estimates at these exposures.

## 4. Discussion

The analyses of the previous sections illustrate a typical modeling conundrum in that the models considered all give differing estimated hazard ratios. For the occupational cohort of the previous section, all examined models provide statistical evidence of elevated risk (or hazard) for carpal tunnel syndrome as SI exposure levels increase relative to unexposed. The linear spline model used by Garg et al. [14] provides perhaps the most compelling evidence of elevated risk of carpal tunnel syndrome at most all exposures as the pointwise estimates of the HR are elevated and significantly larger than one, except for the extreme exposure of 54.0 SI units. A model selection criterion, such as the AIC, can be used to select a single, optimal model, of those considered. Here, the linear spline model is “best” in this sense, but the AIC values for these six models are relatively similar, suggesting general consistency with the data across models. Even though the magnitudes of the point and interval estimates differ between the different models, they are consistent in that they all provide evidence of increased risk with increased exposure except at the highest exposures (compared to a baseline of unexposed), despite the nonsignificant* p* values. Ignoring the effect size evidence, demonstrated in all four of these models, in favor of only the dichotomous results of significance testing would obscure this important information [26].

**Figure 9:**
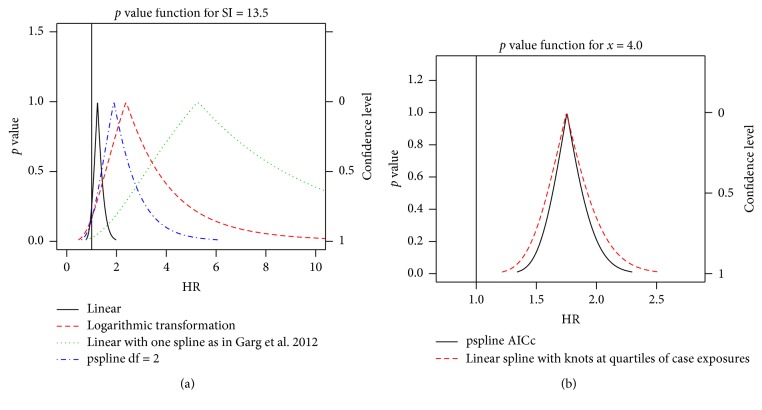
*p* value functions for the risk of carpal tunnel syndrome at an exposure of 13.5 strain index units versus unexposed (a) and for the simulated cohort data at an exposure of* x* = 4.0 versus unexposed (b).

A visual representation of the effect size differences (and similarities) between models can be assessed using the* p* value functions for each model. The* p* value function (as described in chapter 10 of Rothman et al. [27] and in Fraser and Reid [28]) aids in demonstrating similarities and differences based on effect size (*x*-axis) and significance (left *y*-axis) or confidence level (right *y*-axis). Examples of* p* value functions for the carpal tunnel syndrome cohort data and the simulated data are in Figure 9. The null hypothesis hazard ratio of HR = 1 is indicated by the vertical line in each plot and the corresponding *y*-axis value at the which this vertical line crosses a given function gives the* p* value for a two-sided test of this hypothesis. The corresponding confidence interval is defined by the endpoints given by a horizontal line crossing the function at this height. We see that the linear exposure model for the carpal tunnel syndrome cohort suggests a moderate effect size, yet it is more precise when compared to the other methods used, some of which are consistent with large effect sizes. The simulated data example suggests that the penalized spline model and a linear spline model have similar effect sizes. As this is a simulated data set, the magnitude of the effect has no physical meaning, but for the given exposure examined (*x* = 4.0) the estimated effect sizes, while biased, are fairly close to the true HR of 2.0 at this exposure.

One caution when using the spline-based methods was highlighted in Tables 1 and 2 in both the simulated and application data examples. In particular, we noted previously in Sections 2.3 and 3 that the confidence intervals are less precise at the higher exposure values. That is, where the data were sparse there is more variation in the corresponding estimates. In Table 2, this is true even in the linear and logarithmic transformed models, although limited to the highest exposure examined in this table. This can be amplified in survival models with a skewed exposure variable fit using splines as splines have boundary effects [29]. Malloy et al. [18] further emphasized via simulation study that the impact is dependent on the number of observed cases, as opposed to the full cohort size.

**Figure 10:**
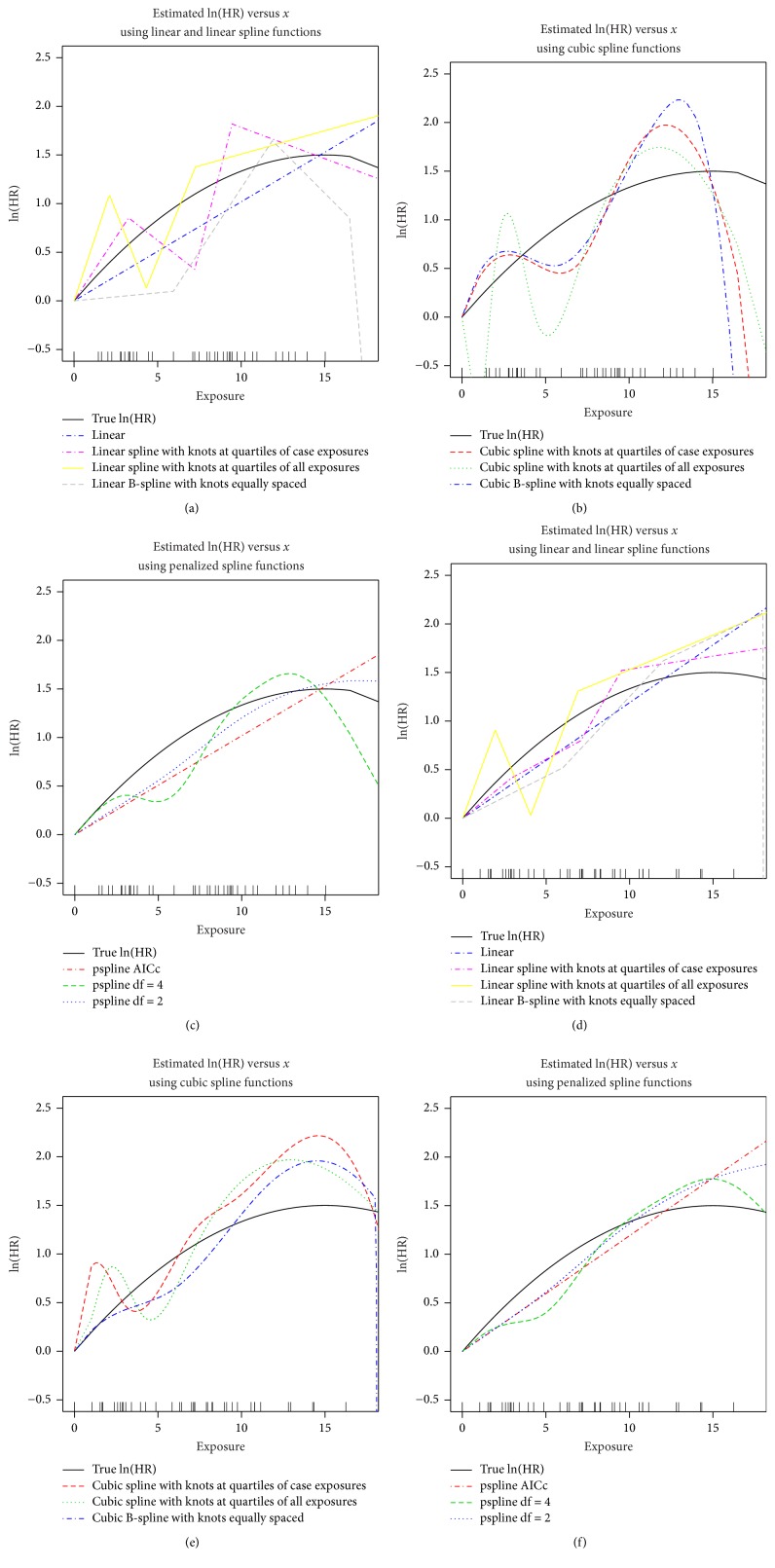
Estimated exposure-response curves on the natural logarithmic scale (logarithm of the hazard ratio) for simulated data with 41 cases in 500 observations (a, b, c) and with 40 cases in 5000 observations (d, e, f) using linear, linear splines, and linear B-splines (a, d), cubic spline and cubic B-splines (b, e), and penalized splines (c, f).

As an illustration, we simulated two new data sets using the simulation set-up of Section 2.1. The first simulated data set is similar to the real-data set of Section 3 and has *n* = 500 individuals and 41 cases. The second data set has *n* = 5000 individuals yet only 40 cases. Estimated exposure-response curves for these two different simulated data sets are given in Figure 10, along with the distribution of case exposures along the *x*-axis. These plots emphasize the impact of the lower number of cases on the estimated curves. The models using basis expansions with linear and cubic splines generally overfit the data, resulting in highly variable estimates across the exposure distribution. In particular, the linear B-spline and the cubic spline fits give estimated hazard ratios (on the logarithmic scale) which decrease substantially after the highest exposed case. The penalized spline fit with the higher degrees of freedom (df = 4 in this case) is similarly variable while the penalized spline with degrees of freedom selected using AICc underfits the data by giving a linear estimate. The penalized spline with df = 2 provides a reasonable estimate to the underlying true hazard ratio. The number of cases is similar for the two data sets (41 and 40, resp.) and thus the fits are also similar, despite an order of magnitude difference in overall sample size (*n* = 500 versus *n* = 5000).

Regression modeling often focuses on interpreting coefficient estimates. When exposure-response relationships are nonlinear and a nonparametric or smoothing method is used to estimate the relationship, the resulting regression coefficients are not interpretable. But, these methods do provide effect size estimates which are interpretable—estimates at specific exposures of interest. The methods illustrated here are easily adapted to include a time-varying exposure. They can also be applied to a covariate of interest which is not an exposure measure but some other quantitative covariates, such as a prognostic factor. In these situations, the reference value of *x* = 0 may not be meaningful, but the methods are equally valid and applicable with other reference values of *x*. The methods described in this paper and other similarly structured smoothing methods can be coded directly, using the enclosed R code as an example. Alternatively, Desquilbet and Mariotti [30] give SAS macro for restricted cubic spline functions and the smooth HR package in R by Meira-Machado et al. [31] implements penalized splines for modeling nonlinearities. Finally, while we illustrate a variety of methods for modeling nonlinear exposure-response relationships, we recommend using these as part of a comprehensive modeling strategy—such as that described in Greenland [32] and Greenland [33]. This should include a diagnostic analysis and assessment of assumptions, paying attention to outliers and influential observations which may impact the functional form [29].

## Figures and Tables

**Table 1:** Estimated hazard ratios (HR) and 95% pointwise confidence intervals from two Cox proportional hazard model fits.

Exposure value *x*	Penalized spline function AICc as in Figure 6	Linear spline function with knots at quartiles of case exposures as in Figure 5(a)	True HR
2.0	1.3 (1.2, 1.5)	1.3 (1.1, 1.6)	1.5
3.0	1.5 (1.3, 1.8)	1.5 (1.1, 2.1)	1.7
4.0	1.8 (1.4, 2.2)	1.8 (1.3, 2.3)	2.0
5.0	2.0 (1.6, 2.5)	2.1 (1.6, 2.7)	2.3
7.0	2.5 (2.0, 3.1)	2.5 (2.0, 3.3)	2.9
9.0	2.9 (2.3, 3.6)	2.9 (2.2, 3.8)	3.5
19.3	3.7 (2.1, 6.3)	4.1 (2.5, 6.5)	4.0
21.1	3.5 (1.7, 7.3)	4.3 (2.5, 7.5)	3.5
24.0	3.3 (1.1, 9.9)	4.7 (2.4, 9.2)	2.6

**Table 2:** Estimated hazard ratios and 95% pointwise confidence intervals from separate Cox proportional hazard models using the carpal tunnel syndrome and strain index exposure data.

Exposure value *x*	Linear	Logarithmic	Linear spline with knot at 13.5	Penalized spline function with df = 2
0.8	1.01 (0.99, 1.03)	1.21 (0.92, 1.59)	1.10 (1.01, 1.20)	1.04 (0.97, 1.11)
6.0	1.10 (0.94, 1.29)	1.88 (0.77, 4.62)	2.09 (1.10, 4.00)	1.35 (0.81, 2.27)
9.0	1.15 (0.91, 1.46)	2.11 (0.73, 6.11)	3.03 (1.15, 7.99)	1.57 (0.78, 3.16)
13.5	1.24 (0.86, 1.77)	2.38 (0.69, 8.19)	5.27 (1.23, 22.57)	1.89 (0.78, 4.60)
18.0	1.33 (0.82, 2.14)	2.60 (0.67, 10.13)	4.85 (1.23, 19.04)	2.12 (0.79, 5.75)
20.3	1.38 (0.80, 2.36)	2.70 (0.66, 11.08)	4.65 (1.23, 17.65)	2.18 (0.78, 6.11)
54.0	2.33 (0.55, 9.84)	3.68 (0.58, 23.35)	2.51 (0.44, 14.42)	2.32 (0.38, 14.15)

## Appendix

### A. Appendix A

The hazard ratio for a given exposure *x* relative to the baseline is

(A.1) $$\begin{array}{c} HR=\frac{\lambda \left(t\;|\;x\right)}{\lambda_{0}\left(t\right)}=\exp\left[\;s\left(\;x\;\right)\;\right]. \end{array}$$

We use a basis expansion representation for *s*(*x*), *s*(*x*) = ∑_*j*=1_^*J*^*b*_*j*_*f*_*j*_(*x*) for *J* known basis functions *f*_*j*_(*x*). Define the *K* by 1 vectors $F(x)={\left[\begin{array}{ccc} f_{1}\left(x\right) & \cdots & f_{J}\left(x\right) \end{array}\right]}^{T}$ and $b={\left[\begin{array}{ccc} b_{1} & \cdots & b_{J} \end{array}\right]}^{T}$. Then *s*(*x*) = *F*(*x*)^*T*^*b* and the estimated log(HR) is $\hat{s}\left(x\right)=F{\left(x\right)}^{T}\hat{b}$. The hazard ratio for an exposure *x*_1_ relative to an exposure *x*_0_ is

(A.2) HR=λt ∣ x=x1λt ∣ x=x0=λ0texp⁡sx1λ0texp⁡sx0=exp⁡sx1−sx0.

This gives an estimated ln(HR) of exposure *x*_1_ relative to an exposure *x*_0_ of

(A.3) log⁡HR^=s^x1−s^x0=Fx1Tb^−Fx0Tb^=Fx1T−Fx0Tb^=Fx1−Fx0Tb^,

where $F\left(x_{1}\right)-F(x_{0})={\left[\begin{array}{ccc} f_{1}\left(x_{1}\right)-f_{1}\left(x_{0}\right) & \cdots & f_{K}\left(x_{1}\right)-f_{K}\left(x_{0}\right) \end{array}\right]}^{T}$. Define this vector of basis function differences as *L* = *F*(*x*_1_) − *F*(*x*_0_) and the estimated exposure-response can be written as $\hat{s}\left(x_{1}\right)-\hat{s}\left(x_{0}\right)=L^{T}\hat{b}$. A (1 − *α*)100% confidence interval for the log(HR) has the form $L^{T}\hat{b}\pm z_{1-\alpha /2}\sqrt{L^{T}Var\left(\hat{b}\right)L}$, where *z*_1−*α*/2_is the 1 − *α*/2 cut-off from a standard normal distribution, $Var\left(\hat{b}\right)$ is the estimated variance-covariance matrix of the estimated coefficients $\hat{b}$, and $\sqrt{L^{T}Var\left(\hat{b}\right)L}$ is the standard error of the estimated log(HR). The coefficient estimates, $\hat{b}$, and the corresponding variance-covariance matrix, $Var\left(\hat{b}\right)$, are generally output by standard software packages. A corresponding confidence interval for the HR can be found either by employing the delta-method (such as that given on p. 58 of Lehmann and Casella [34]) or by following the advice of Collett [35] who notes that the distribution of the estimate of the ln(HR) is closer to a normal distribution than the distribution of the estimate of the HR and thus suggests exponentiating the confidence interval for the ln(HR).

The linear truncated power basis coefficients estimates have a nice interpretation in terms of the estimated change in the slope of the exposure-response curves that occurs at the knot points. For instance, the estimated slope for exposures up until the first knot point of 3.0 corresponds to the coefficient ${\hat{b}}_{1}=0.1335.$ The slope is estimated to change at an exposure of 3.0 by ${\hat{b}}_{2}=0.0260$ and remain at ${\hat{b}}_{1}+{\hat{b}}_{2}=0.1595$ up until an exposure of 5.5 at which point the slope is estimated to change by ${\hat{b}}_{3}=-0.0692$ and remain at the resulting estimated slope of 0.0903 up until an exposure of 8.3. At 8.3, the slope is estimated to further decrease by ${\hat{b}}_{4}=-0.0581$ to 0.0322 and stay at this slope for the remaining exposures. For any given exposure, the estimated hazard of an event is relative to a reference exposure.

The R script for creating the linear truncated power basis using knots at the quartiles of the case exposures is given in Appendix B. This is based on input data of the form (*x*, *c*, *t*) which corresponds to the exposure variable (*x*), event or censoring indicator (*c* = 1 if the event of interest occurred; otherwise it is 0), and the observed or censored survival time,* t*. For the plots to display meaningfully, the data must be presorted on the *x*-variable.

### B. Appendix B

The R script for creating the linear truncated power basis and fitting the corresponding Cox PH model is as follows:

# invoke the survival package
# it must have been previously downloaded
library(survival)
# data are (x,t,c)
# find quartiles of cases
q1=quantile(x[c==1])[2]
q2=quantile(x[c==1])[3]
q3=quantile(x[c==1])[4]
# create the linear truncated power basis with three knots
lin.spline.basis = matrix(nrow=n,ncol=4)
lin.spline.basis[,1] = x
lin.spline.basis[,2] = x-q1
lin.spline.basis[lin.spline.basis[,2] < 0,2] = 0
lin.spline.basis[,3] = x-q2
lin.spline.basis[lin.spline.basis[,3] < 0,3] = 0
lin.spline.basis[,4] = x-q3
lin.spline.basis[lin.spline.basis[,4] < 0,4] = 0
# fit the Cox PH model
coxout = coxph(Surv(t,c)~lin.spline.basis,na.action=na.omit,
  method="breslow")
# the default fitted values have mean(x) as the reference
fitloghr = predict(coxout)
# when plotted the default is "mean" shifted
# note that the data must be sorted based on the x-variable for
# plots to display correctly
plot(x,fitloghr,type= ＇l＇,ylim=c(-1,2),lwd=2)

The R script for computing fitted values at each exposure value, their corresponding standard errors, pointwise 95% confidence intervals, and plotting the results is as follows:

# fitted values with x = 0 as the reference exposure
L = t(lin.spline.basis)
b = coxout$coef # estimated b coefficients
loghr = t(L) %∗%b # these are the fitted values at each value of x
varb = coxout$var # extract the variance estimates
varLb = t(L)%∗%varb%∗%L # the corresponding variance - covariance matrix # for the fitted values
SELb = sqrt(diag(varLb)) # the corresponding standard errors
# now we can create a 95% confidence interval
lower = loghr-1.96∗SELb
upper = loghr+1.96∗SELb
# plot the results
plot(x,loghr,type= ＇n＇,ylim=c(0.0,2.8),xlab="exposure",
  ylab="estimated log(HR)",main= ＇log(HR) vs. x＇)
mtext(＇using linear spline basis with knots at quartiles of case
  exposures＇)
polygon(c(rev(x),x), c(rev(upper), lower), col=＇grey80＇, border=NA)
lines(x,upper,col=1,lty=2)
lines(x,lower,col=1,lty=2)
lines(x,loghr,lwd=2)
legtxt = c("upper and lower 95% CI","estimate")
legend(0,2.8,legtxt,lty=c(2,1),lwd=c(1,2))

### C. Appendix C

The R script for fitting a penalized spline with the degrees of freedom selected using the AICc is below. It assumes the survival() package is installed and loaded. The data are of the form: x = exposure variable, t = survival times, c = event/case indicator. Fixed degrees of freedom can be used by replacing the df=0 option with the desired degrees of freedom, say df=2 for a two-degree-of-freedom penalized spline. When using a set of degrees of freedom, the user should also then delete the caic=T option.

coxout.aicc = coxph(Surv(t,c)~pspline(x,df=0,caic=T),data=subdata, na.action=na.omit,method="breslow")
print(coxout.aicc)

The corresponding output from the print() command gives the chi-square test for the nonlinearity on the second line of the output, here having an observed test statistic value of 11.3 on 1.86 degrees of freedom, yielding a* p* value of 0.003. Note also that the first line of the output gives an estimate of the linear coefficient, here 0.0902, with a standard error of 0.00899.

**Table:**

Call:						
coxph(formula = Surv(t, c) ~ pspline(x, df = 0, caic = T), data = subdata, na.action						
= na.omit, method = "breslow")						
	coef	se(coef)	se2	Chisq	DF	p
pspline(x, df = 0, caic =	0.0902	0.00899	0.00898	100.8	1.00	0.000
pspline(x, df = 0, caic =				11.3	1.86	0.003
Iterations: 10 outer, 29 Newton-Raphson						
Theta= 0.996						
Degrees of freedom for terms= 2.9						
Likelihood ratio test=116 on 2.86 df, p=0 n= 5000						

### D. Appendix D

Formal tests can also be evaluated for the truncated power basis methods. As the truncated power bases include a linear term in their expansion, this corresponds to testing the null hypothesis Ho: *b*_2_ = *b*_3_ = ⋯ = *b*_*p*+*K*_ = 0 versus the alternative hypothesis that at least one of these coefficients is nonzero. That is, Ho: *s*(*x*) = *b*_1_*x* versus Ha: *s*(*x*) = *b*_1_*x* + *b*_2_*x*^2^ + ⋯+*b*_*p*_*x*^*p*^ + *b*_*p*+1_(*x* − *k*_1_)_+_^*p*^ + ⋯+*b*_*p*+*K*_(*x* − *k*_*K*_)_+_^*p*^. A likelihood ratio test can be derived to test this “reduced” model in Ho versus the “full” model in Ha. The test statistic has the form:

(D.1) X=−2logL^reducedL^full=−2logL^reduced+2logL^full,

where $\hat{L}$ denotes the maximized (partial) likelihood function evaluated at its corresponding maximum likelihood coefficient estimates. These are evaluated at the estimates from both the reduced model, $\hat{L}$  (reduced), and the full model, $\hat{L}$  (full). This test statistic asymptotically has a chi-square distribution with the degrees of freedom corresponding to the difference in the number of coefficients between the full and reduced models. It is necessary that the reduced model is nested within the full model and can be obtained by constraining coefficients in the full model.

The coxph() function in the survival package provides the maximum log likelihood values for the fitted model and the null model. R commands and the corresponding output for formally testing the nonlinear component of the linear truncated power basis in Figure 5 are given below. From this, we see that $log\left[\hat{L}(\text{reduced})\right]=-6588.089$ and $log\left[\hat{L}\left(\text{full}\right)\right]=-6580.466$ giving a test statistics of* X* = 15.28619 and corresponding* p* value of 0.0016, based on a chi-square distribution with 3 degrees of freedom (the difference in the number of coefficients between the two models). Thus, there is evidence of a nonlinearity in the exposure-response relationship. The likelihood ratio test can also be used as an alternative test of the nonlinearity in the penalized spline models.

The R code and corresponding output for the likelihood ratio test of nonlinearity in the linear truncated power basis expansion are as follows:

> coxout.lin.spline = coxph(Surv(t,c) ~ lin.spline.basis, na.action = na.omit, method="breslow")
> coxout.lin.spline
Call:
coxph(formula = Surv(t, c) ~ lin.spline.basis, na.action = na.omit, method = "breslow")

**Table:**

	coef	exp(coef)	se(coef)	z	p
lin.spline.basis1	0.1441	1.155	0.0219	6.593	4.3e-11
lin.spline.basis2	-0.0796	0.923	0.0402	-1.979	4.8e-02
lin.spline.basis3	-0.0258	0.975	0.0746	-0.346	7.3e-01
lin.spline.basis4	-0.4210	0.656	0.3790	-1.111	2.7e-01

Likelihood ratio test=118 on 4 df, p=0 n= 5000, number of events= 804
> coxout.lin. spline$loglik [2]
[1] -6580.446
> coxout.lin = coxph(Surv(t,c) ~ x,
data=subdata,na.action=na.omit,method="breslow")
> coxout.lin
Call:
coxph(formula = Surv(t, c) ~ x, data = subdata, na.action = na.omit, method = "breslow")

**Table:**

	coef	exp(coef)	se(coef)	z	p
x	0.0882	1.09	0.00828	10.7	0

Likelihood ratio test=102 on 1 df, p=0 n= 5000, number of events= 804
> coxout.lin$loglik[2]
[1] -6588.089
> X = -2∗(coxout.lin$loglik[2]-coxout.lin. spline$loglik [2]) # test statistic
> pval = 1-pchisq(X,3)
> data.frame(test.stat=X,pvalue=pval)

**Table:**

	test.stat	p value
1	15.28619	0.001587718
